# Synthesizing intelligible utterances from EEG of imagined speech

**DOI:** 10.3389/fnins.2025.1565848

**Published:** 2025-04-17

**Authors:** Wenjing Xiong, Lin Ma, Haifeng Li

**Affiliations:** Faculty of Computing, Harbin Institute of Technology, Harbin, China

**Keywords:** brain-computer interface, deep neural network, dynamic time warping, partial feedback, spectral subtraction

## Abstract

Decoding natural language directly from neural activity is of great interest to people with limited communication means. Being a non-invasive and convenient approach, the electroencephalogram (EEG) offers better portability and wider application potentiality. We present an EEG-to-speech system (ETS) that synthesizes audible, and highly understandable language by EEG of imagined speech. Our ETS applies a specially designed X-shape deep neural network (DNN) to realize an End-to-End correspondence between imagined speech EEG and the speech. The system innovatively incorporates dynamic time warping into the network’s training process, using actual speech EEG data as a bridge to temporally align imagined speech EEG signals with speech signals. The ETS performance was evaluated on 13 participants who pretraining four Chinese disyllabic words. These words cover all four tones and 40% of the phonemes in Chinese. Our synthesized utterances’ average accuracy across all subjects was 91.23%, the average MOS value was 3.50, and the best accuracy was 99% with an MOS value of 3.99. Furthermore, a partial feedback mechanism for DNN and spectral subtraction-based speech enhancement are introduced to improve the quality of generated speech. Our findings prove that non-invasive approaches can be a significant step in regaining verbal language ability.

## 1 Introduction

Language and cognition are separate and closely related mechanisms of the mind (Perlovsky, 2009). For people who are unable to speak due to physical or neurological impairments, the significance of synthesizing natural speech through brain-computer interfaces is enormous (Pandarinath et al., 2017; Brumberg et al., 2018; Koch et al., 2019). In particular, non-invasive and highly reliable signal acquisition and synthesis techniques are more significant to patients (Wöstmann et al., 2017). In this paper, we tried to explore the technology based on electroencephalogram (EEG) to synthesize imagined Chinese speech.

The brain processes information about one’s own speech from three different sources (Hickok and Poeppel, 2004): (1) overt speech, which is directly vocalized speech. (2) silent articulation, which refers to vocal organs such as the mouth, tongue, and throat that are involved in movement but do not produce sound. (3) covert speech, also known as imagined speech or inner speech, in which the vocal organs do not move and are silent. The speech-processing cortical network of the brain consists of ventral and dorsal neural pathways that process semantic and articulatory representational information, respectively (Panachakel and Ramakrishnan, 2021). Several studies based on different neuroimaging data have shown that the bilateral superior temporal gyrus is an important locus for speech information processing (Klein et al., 2001; Gu et al., 2013; Ge et al., 2015; Kwok et al., 2017). Combining the surface impoverished hypothesis proposed by Oppenheim and Dell (2008), Brocklehurst and Corley (2011) research on the inner speech of people who stutter, and the monitoring results of brain activity by different BCI (Bocquelet et al., 2016; Cogan et al., 2014; Huang et al., 2002; Stephan et al., 2020), showed neural correlates of three self-speech, that they are shown to activate overlapping brain regions. Previous studies have shown that overt speech, covert speech, and silent articulation activate overlapping brain regions, particularly in the motor and premotor cortices. However, the extent to which these processes rely on a shared neural network versus distinct pathways remains a topic of ongoing debate, with some studies suggesting graded activation differences while others propose separate mechanisms.

In recent years, there have been several studies that applied neural activity signals to synthesize natural speech. Guenther et al. (2009) synthesized imagined speech by implanting a single Neurotrophic electrode in the aphasic patient’s left precentral gyrus, synthesizing five English vowels. Anumanchipalli et al. (2019) were the first to synthesize fluent natural language from neural activity signals, successfully using ECoG to construct a mapping between overt speech neural activity and vocal motor trajectory based on LSTM and HMM methods. And achieve an average word error rate (WER) (Ali and Renals, 2018) of 3%. Angrick et al. (2019) also used the ECoG signal to construct a mapping from overt speech neural activity to the Mel-spectrogram of speech based on the CNN method, and then the Mel-spectrogram can be converted to speech by a WaveNet (Oord et al., 2016) vocoder. It was the first to achieve the synthesis of high-quality audible speech. Two other latest studies applying imagined speech neural activity to synthesize natural speech are both from implanted electrodes. Angrick et al. (2021) applied Stereo-Electroencephalography (sEEG) to synthesize silent articulation and imagined speech in real-time. Moses et al. (2021) developed a neural prosthesis device to filter suitable words based on a speech detection model and a word classifier, while a long sentence was decoded based on the Viterbi algorithm (Forney, 1973), and finally, the speech was synthesized from the brain activity of a paralyzed patient who could not vocalize. However, considering that the target group for applying measured neural activity to synthesize natural speech is aphasic patients, it is strongly necessary to apply silent articulation or imagined speech neural activity to synthesize natural speech. Nieto et al. (2022) proposed an experimental paradigm based on silent articulation and imagined speech, and opened the EEG dataset of the Dutch language collected under this experimental paradigm, hoping to promote research on the synthesis of natural speech based on EEG and other non-invasive brain-computer interfaces. Radford et al. (2018) based on the fMRI signal from the subject’s listening task which combined with a large language model (LLM) to predict the subject’s brain activity from the perspective of semantic decoding (Tang et al., 2023). fMRI temporal lag was effectively solved by LLM, but there was a discrepancy between semantic accuracy and lexical accuracy. It could not accurately decode the speech and could not cross-subject. Firstly there is a lack of research on synthesizing speech directly from neural activity, and secondly, there is no synthesis of imagined speech through non-invasive brain-computer interfaces.

In terms of the linguistic aspects, for a tonal language like Chinese (Duanmu, 1990), different pitch patterns will represent different lexical meanings. In contrast, in non-vocalic languages such as English, pitch changes are not complex and do not convey lexical information (Cheng, 1968). And Chinese contains more homophones compared to English, which makes Chinese more dependent on pitch and context when conveying information. Also, considering the differences in processing patterns of phonology in the brain across languages creates migration barriers in the application of brain activity signals to synthesize Western speech and Chinese speech methods (Ge et al., 2015). Lopez-Bernal et al. (2022) summarize the advancements in using EEG for decoding imagined speech, focusing on the classification of imagined articulation of English words or morphemes, while highlighting the absence of direct end-to-end synthesis of speech.

We applied imagined speech EEG to generate Chinese speech, the obstacle is that EEG has the natural disadvantage of insufficient spatial resolution and temporal accuracy compared to sEEG, ECoG, and fMRI (Bookheimer et al., 1995). However, the biggest challenge is the EEG is highly susceptible to a variety of noise and artifacts: ocular artifacts (Croft and Barry, 2000) and myogenic artifacts (Muthukumaraswamy, 2013). So the EMG artifacts caused by the oral movements of the subject due to open speech and unavoidable blinking can form a highly intrusive and complex noise. If only the EEG signal of the subject’s imagined articulation is collected, how to align the signal with the subject’s actual articulated speech is a challenge urgent to be solved (Schultz et al., 2017).

This paper designed an experimental paradigm to separate the articulation task from the imagined articulation task, which compensates for the disadvantage of EEG being susceptible to myoelectric interference. We proposed an EEG-to-speech system, achieving the application of imagined articulation of EEG to synthesize audible and intelligible Chinese speech, which has strong exploration significance.

## 2 Material and methods

### 2.1 Experimental design

We provided an experimental paradigm for the asynchronous capture of imagined speech EEG and speech. The experiment corpus contains the following four words: “脑电/nao3 dian4/,” “合成/he2 cheng2/,” “中文/zhong1 wen2/,” and “语音/yu3 yin1/.”

**FIGURE 1:**
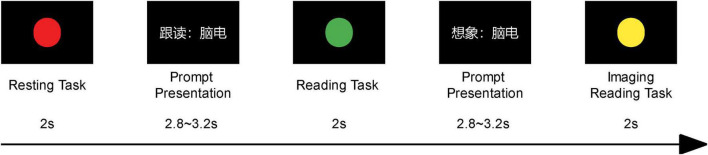
The flow chart of a single trial. The above flow chart shows the screen images that the subjects could see in a trial in chronological order, and the subjects will complete the corresponding tasks according to the stimulus content (different colored circles). The description and duration of each screen are shown at the bottom of the picture.

There were 15 blocks in every experiment, separated by a 10-s pause. Each block contains 2 × 4 (corresponding to four words) trials, the stimulus presentation duration was two seconds. That is, there are 15 trials for each word, for a total of 60 trials for each subject. In a single trial, we designed three tasks, which were: the resting task, the reading task, and the imaging reading task. For each task, we prompted the subjects to complete the corresponding task through different colored circles, and the flow of the trial using the term “nao dian” is shown in Figure 1. Moreover, since the primary visual cortex (V1) contains two types of color-sensitive neurons that are responsive to different wavelengths of visible light in the spectrum, the processing of traffic light stimuli can be completed without requiring further processing in higher-level visual areas (Shapley and Hawken, 2011). The subjects were given instructions and took pre-training before the experiment started. Subjects were instructed to look at the “+” symbol in the middle of the screen, maintain focus, and respond rapidly after the stimulus was presented in each block.

This color-coded design was essential for clearly defining task conditions and ensuring better control over participants’ cognitive and neural activities. Additionally, presenting the prompt before the signal light ensured that participants always viewed the same visual stimulus during both overt and covert speech tasks, thereby minimizing interference from the visual cortex and improving the precision of auditory cortex activity localization.

Regarding potential EEG influences, primary visual cortex (V1) neurons are known to respond selectively to different wavelengths of visible light, allowing participants to process the signal lights rapidly without deeper visual processing. This design enhances task clarity while reducing visual processing interference in speech-related neural activity.

### 2.2 Subjects

16 individuals in all were enrolled. All subjects were right-handed according to the Sharpshooter Scale test (Snyder and Harris, 1993). Due to the disorganized spontaneous brain waves of some subjects, data were finally collected from 13 valid subjects (5 females and 8 males, all aged 22 to 28). They had good health, no neurological or mental illnesses, and spoke Chinese as their native language, and English as their first foreign language, according to the questionnaire results. Both their corrected eyesight and hearing are normal.

### 2.3 Data preprocessing

The EEG recordings used a Neuroscan Synamps2 Amplifier with a bandpass filter ranging from 0.05 Hz to 150 Hz, sampling at 1,000 Hz. Electrodes were placed according to the international 10/20 system, using a 64-channel Electro-Cap (Compumedics Neuroscan) with scalp impedance under 5 kΩ. The speech was concurrently recorded via a separate microphone at 44,100 Hz, down-sampled to 16,000 Hz, and pre-processed with a frame of 400 ms and frame-shifting of 80 ms, employing a Hamming window function. All EEG recordings were conducted in a controlled laboratory environment with minimized external auditory and electromagnetic noise.

**FIGURE 2:**
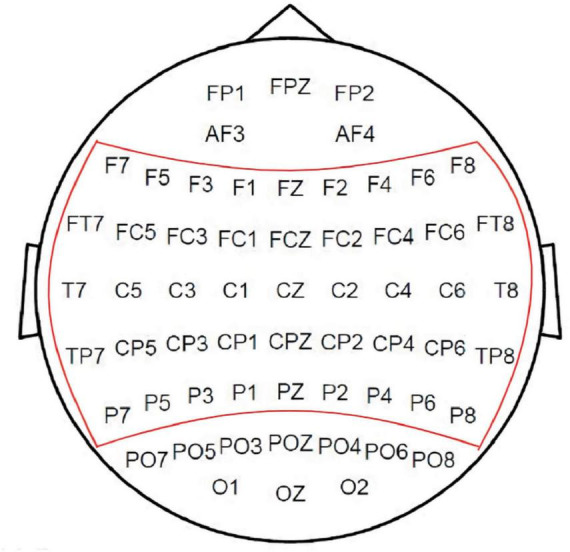
Electrode positions for the 45 channels screened in the experiment. Relative positioning of the 64 electrode channels on the scalp, with the 45 channels selected for analysis encircled by the red line.

Before analysis, EEG data underwent noise reduction and the selection of 45 electrode channels, distributed across the scalp as depicted in Figure 2. A Chebyshev bandpass filter (1–45 Hz) was then applied. We selected 45 electrodes based on their relevance to speech-related brain regions, primarily covering the frontal, central, and temporal areas to maximize signal quality while reducing redundancy. Additionally, the 1–45 Hz frequency range was chosen to eliminate 50 Hz power line interference and capture key neural oscillations associated with cognition and motor processes.

EEG segmentation was based on the stimulus presentation event onset, resulting in 120 segments per subject. The first 1,000 ms post-event served as baseline correction, followed by framing and windowing operations similar to those applied to speech data. It is pertinent to note that the inputs to our network, whether derived from speech or EEG signals, are characterized by their temporal waveform amplitudes.

### 2.4 System modeling

We constructed an EEG-to-speech system abbreviated as ETS to implement the application of EEG to synthesize imagined speech. The system consists of two X-shaped neural network models for data preparation and multimodal representation learning, respectively. Notably, the network incorporates specialized fusion modules at different stages, optimizing performance through the integration of dynamic time warping, spectral subtraction, and other machine learning algorithms during both training and testing phases. The proposed X-shaped model is an adaptation of standard fully connected networks, incorporating a crossover structure to enhance feature integration. While similar architectures exist in other domains, this design is tailored for EEG-based speech synthesis. Its main advantage lies in its ability to effectively combine features from different processing pathways, potentially improving representation learning for EEG signals.

**FIGURE 3:**
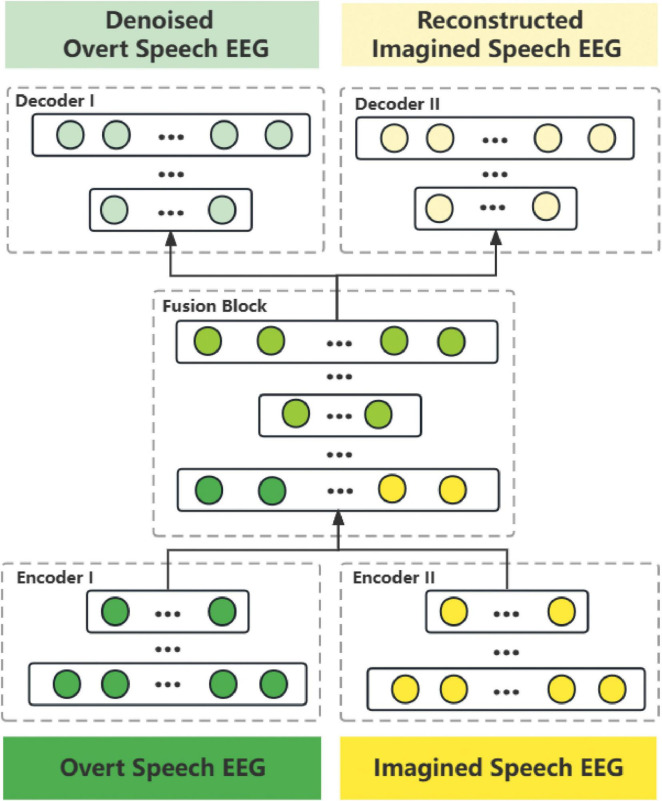
Constructing temporally aligned speech and EEG based on the XI model. The data set is identified by the curly brace, and time-aligned data pairs are identified by the parenthesis. (1) Union the overt speech EEG and the imagined speech EEG by the XI model for denoising. (2) Using DTW to align the timing of the imagined speech EEG and the denoised overt speech EEG. (3) Based on the overt speech EEG (before denoising) and speech is naturally aligned in timing, aligning imagined speech EEG with speech by overt speech EEG. (4) Judging whether the altered overt speech EEG quality meets the required level. If not, repeat steps 1–3, otherwise, output the paired data. The assessment of altered overt speech EEG quality was based on an empirically determined loss threshold.

**FIGURE 4:**
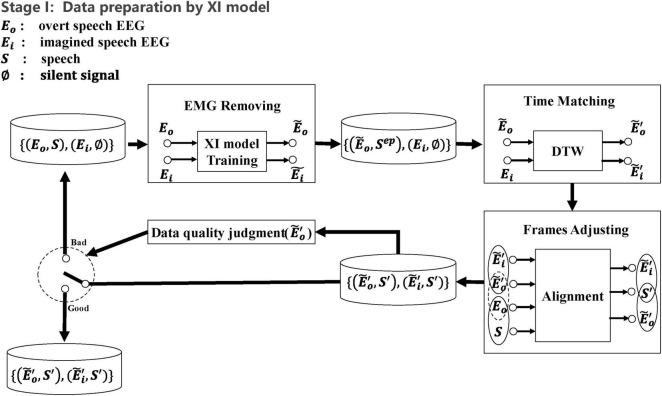
Network structure diagram of the XI model. It consists of two encoders, a fusion block, and two decoders. The non-fusion modules (individual encoders and decoders) compress and expand features with a ratio of 30%, while the fusion block applies a more aggressive 75% compression-expansion ratio to enhance cross-modal feature integration. The fusion block, designed as a symmetric structure, iteratively compresses and reconstructs multimodal features, promoting greater information exchange between network paths.

The major goal of the system is to synthesize speech using imagined speech EEG, but in practice, it is impossible to gather imagined speech EEG that strictly matches the speech in the temporal domain. It should be observed, though, that there is a natural alignment between the synchronous collected speech and overt speech EEG. Therefore, we propose the first X-shaped model (named XI model) for aligning overt speech EEG and imagined speech EEG in the time domain to obtain paired speech and imagined speech EEG. We designed a novel training method (see Figure 3) that automatically removes EMG artifacts from the overt speech EEG while completing the EEG alignment. We pre-trained the XI model by using the imagined speech EEG added with white noise to simulate the overt speech EEG. The learning rate and batch size used during pre-training were the same as those used in the formal training of the XI model, specifically a learning rate of 0.001 and a batch size of 420. The signal-to-noise ratio (SNR) was initially set to 0 during pre-training. This choice was made to simulate a scenario where the imagined speech EEG is masked by noise, thus providing a challenging environment for the model to differentiate weak signals from noise. By setting the SNR to 0, we encourage the model to focus on extracting features even in the worst-case scenario, which enhances its robustness. The noise was artificially generated and introduced at the preprocessing stage. This enables the network to quickly enter the working state in the formal training process to complete the task of denoising the overt speech EEG. The construction of the XI model is based on the Dynamic Time Warping (DTW) algorithm (Dynamic Time Warping (DTW) 2007), Deep Autoencoder (Lange and Riedmiller, 2010), and Multimodality Fusion Learning (MFL) (Baltrusaitis et al., 2019). The network structure of the XI deep autoencoder is shown in Figure 4. The decoder on both sides of this network expects output were imagined speech EEG (adjusted the frame sequence according to the DTW every time), and the loss function is the mean square error.

In terms of model parameter details, the dual input of the XI model matches the scale of 45-channel, 400 ms EEG data, resulting in an input layer neuron scale of 18,000. Apart from the fusion module, the neuron scale decreases by 0.75 proportionally with network depth. The fusion module also follows a compression-expansion scheme with a 0.75 ratio. The network comprises 9 layers from input to output, with all activation functions set to tanh. The loss function is mean squared error (MSE), optimized using Adam optimizer with a learning rate of 0.001. The batch size is set to 21*20 (2 s of EEG data could be segmented into 21 frames with an 80 ms frameshift). To prevent overfitting during network training, we employed an early stopping mechanism based on the comprehensive evaluation of two criteria: (1) the number of training iterations, capped at a maximum of 10,000 epochs, and (2) the regression mean difference between the network output EEG and the target imagined speech EEG. Specifically, the training process was terminated when the regression mean difference failed to improve for 50 consecutive epochs, indicating convergence. This approach ensured that the model was trained with optimal performance while avoiding overfitting to the training dataset. By incorporating this stopping criterion, we achieved a balance between minimizing training error and maintaining generalization capability on unseen data.

**FIGURE 5:**
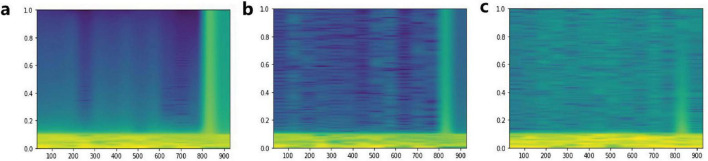
Comparison of noise reduction performance between XI model and ICA. Graph **(a)** is the spectrogram of imagined speech EEG. Graph **(b)** is the spectrogram of overt speech EEG denoised by the XI model. Graph **(c)** is the spectrogram of overt speech EEG denoised by ICA.

In addition, we compared the noise reduction ability of the XI model with the Independent Component Analysis (ICA) (Delorme and Makeig, 2004), and the results are shown in Figure 5 and Table 1. The source of the data in Table 1 is measured by calculating the mean Pearson correlation coefficient (PCC) (Benesty et al., 2009) on the spectrogram between the noise-reduced overt speech EEG and the imagined speech EEG from the same trial. The *t*-value for the word “He Cheng” is notably high (56.90), likely due to the particularly stable PCC distribution in the XI model, resulting in lower within-group variance. This stability leads to a lower variability in the results, reinforcing the robustness of the model. Despite this high *t*-value, the significant improvement in PCC remains statistically valid, consistent with the improvements observed for other words. The results of the *t*-test confirm that the XI model shows a significant improvement in noise reduction compared to ICA, as evidenced by the low *p*-values (e.g., 1.16e-21 for “Yu yin”).

**TABLE 1:** Mean PCC between denoised overt speech EEG and imagined speech EEG.

Word	ICA (PCC+Std)	XI model (PCC+Std)	*t*-test*t*-value, *p*-value
Nao dian	0.44 ± 0.05	0.53 ± 0.04	6.93, 5.76e-11
He cheng	0.43 ± 0.04	0.89 ± 0.01	56.90, 2.38e-72
Zhong wen	0.43 ± 0.06	0.57 ± 0.05	10.94, 7.99e-18
Yu yin	0.43 ± 0.05	0.58 ± 0.04	12.41, 1.16e-21

**FIGURE 6:**
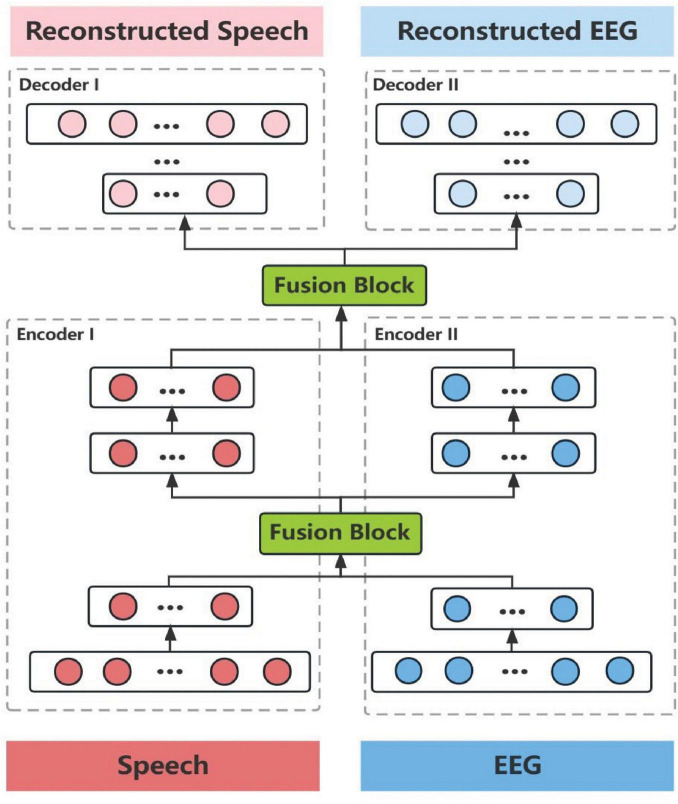
Network structure diagram of the XII model. It consists of two encoders, two fusion blocks, and two decoders. The progressive fusion strategy operates in a staged manner, incorporating fusion blocks at multiple feature levels. Non-fusion modules (encoders and decoders) apply a 30% compression-expansion ratio, while the fusion blocks utilize a 75% compression-expansion ratio to maximize cross-modal integration. Data from both modalities are first fused at the lower-level features, where detailed information is abundant, and subsequently fused again at higher-level features after compressed encoding. This structure enables early-stage fusion, preserving fine-grained modality-specific details while progressively aligning higher-level representations. This design supports richer cross-modal interactions, enhancing the formation of comprehensive shared representations.

**FIGURE 7:**
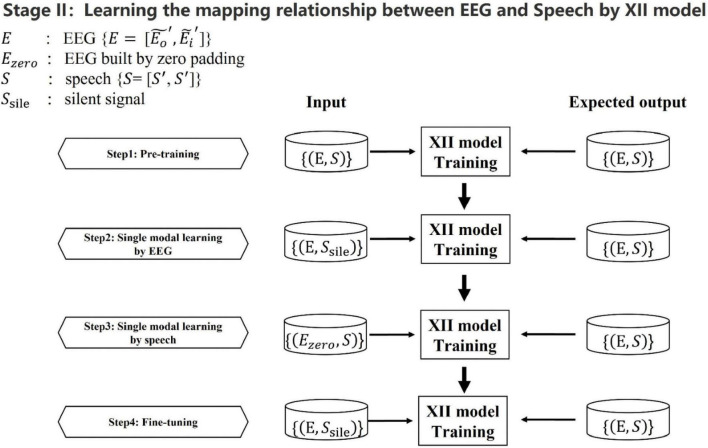
Learning the mapping relationship between EEG and speech based on the XII model. The speech and EEG presented here were created by the XI model which are temporally aligned. The silent speech and masked EEG are created by zero padding. We sequentially create the following four input combinations during the training process: EEG and speech, EEG and silent speech, masked EEG and speech, and EEG and silent speech. All of these four input pairs’ expected outputs are EEG and speech. Zero padding was applied to create masked data, enabling the model to enhance cross-modal learning by reconstructing both modalities from unimodal input. This step was incorporated during training to improve the mutual information between EEG and speech representations, thereby strengthening their shared feature space.

After training the XI model and aligning the outputs using the DWT algorithm, we obtained two types of time-domain aligned data pairs: speech paired with denoised overt speech EEG, and speech paired with imagined speech EEG. Next step, we based on the second X-shaped model (named XII model), to learn the corresponding relationship between the speech frames and the EEG frames built by the XI model. Since EEG data contains both temporal information of speech information processing and spatial information of the brain, it is richer and more complex than speech information alone. Simply combining the lowest-level unimodal features cannot create the appropriate shared representation of these two modal data. In this paper, a progressive fusion strategy was proposed to construct The XII model. The XII model architecture is shown in Figure 6, and the training strategy is shown in Figure 7. Training and test data were strictly separated within each participant to prevent data leakage, following standard within-subject EEG classification protocols.

In the XII model, the dual input corresponds to 18,000 data points for the 400 ms EEG signal and 6,400 data points for the 400 ms speech signal. Apart from the fusion module, the neuron scale decreases by 0.75 proportionally with network depth. The fusion module also follows a compression-expansion scheme with a 0.75 ratio. The network comprises 12 layers from input to output, with all activation functions set to tanh. The loss function is mean squared error (MSE), optimized using Adam optimizer with a learning rate of 0.001. Batch size is set to 21*20.

In the training process of the XII model, we refined the adjustment of the network’s training state by employing different early stopping strategies for its stages. For the first three stages, the early stopping mechanism was consistent with that of the XI network, where a combination of training iterations and loss convergence was used as the evaluation criterion. However, in the fourth stage, the strategy was adjusted to focus on the signal quality. Specifically, speech synthesis signal-to-noise ratio (SNR) evaluations were conducted every 1,000 epochs, for a total of 20 evaluations. The network parameters corresponding to the lowest average SNR across these evaluations were selected as the final result, ensuring optimal performance in this critical stage.

**FIGURE 8:**
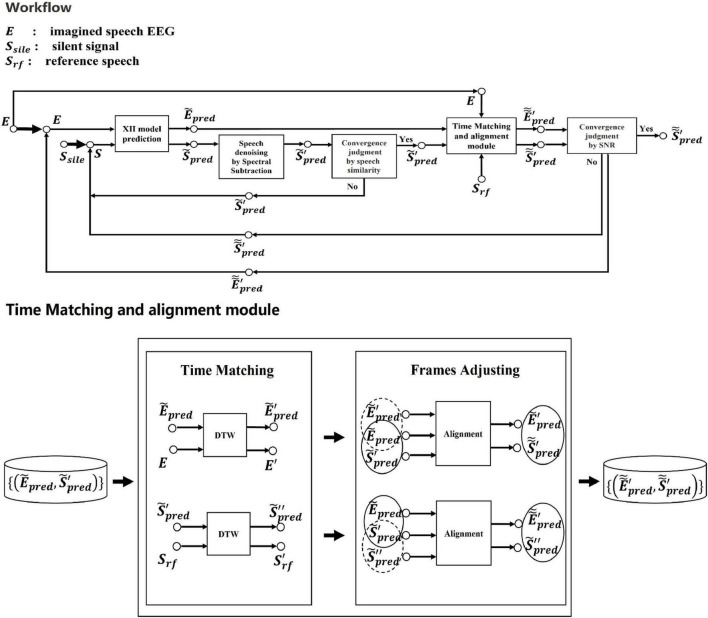
Flowchart for applying the XII model from the ETS system to synthesize speech. The XII model receives imagined speech EEG as input and produces synthesized speech as the final output. (1) Create the silent speech signal, transmit it to the XII model with EEG for prediction, and then use spectral subtraction to eliminate the noise in the predicted speech. (2) If the denoised predicted speech does not converge with the input speech of the XII model (Measuring similarity by PCC), send the denoised speech and EEG pairing back to the network for prediction and repeat steps 1–2. If not, the denoised speech and predicted EEG are delivered to the time matching and frame adjustment module for timing correction. (3) If the corrected speech’s signal-to-noise ratio is below the threshold, the predicted EEG will be sent to the XII model for prediction along with the corrected speech, repeat steps 1–3. If the threshold value is reached, the speech is synthesized and output. As for the time matching and alignment module, the predicted EEG and the denoised predicted speech are first aligned based on DTW by EEG and reference speech, and then the EEG and speech are aligned according to the frame correspondence after adjusting the timing, respectively.

In the application stage of the ETS system, we combined the partial feedback mechanism (Li et al., 2019) with DTW, and the noise of the reconstructed speech is removed by spectral subtraction (Boll, 1979), which effectively improves the quality of the synthesized speech, The specific process is shown in Figure 8. We assume that the generated speech during the initial and final 200 ms intervals of the 2-s synthesis comprises silence. Consequently, we use the signals from these segments of the synthesized speech as noise signals, estimate their spectra, and then extend this estimation to the entire 2-s duration. This approach enables us to optimize spectral subtraction for the synthesized speech.

## 3 Results

A total of four disyllabic Chinese words were selected for the experimental material: “脑电/nao3 dian4/,” “合成/he2 cheng2/,” “中文/zhong1 wen2/,” and “语音/yu3 yin1/,” which means “EEG,” “synthesis,” “Chinese,” and “speech.” These words cover all four tones and 13 phonemes of the total 32 ones in the Mandarin language.

Speech synthesis quality evaluation methods are mainly divided into objective and subjective perspectives (Wagner et al., 2019). The objective perspective is judged by directly calculating the similarity between the synthesized speech and the reference speech in the frequency domain and Mel spectrogram, and the calculation includes time distortion to align the two speech signals (in the case of different speech times), based on Euclidean distance, Pearson correlation coefficient and other distance methods to calculate the similarity. Reference speech are manually selected from recorded speech with high quality. The subjective perspective is to evaluate speech quality by employing questionnaires, explicitly asking users for their impression of various quality dimensions. Such as mean opinion score (MOS) (Viswanathan and Viswanathan, 2005) and multiple stimuli with hidden reference and anchor (MUSHRA) (2015). To present the quality of the synthesized speech, we first calculated the Pearson correlation coefficients between the synthesized speech and the reference speech on the spectrograms and Mel-scale Frequency Cepstral Coefficients (MFCC). Meanwhile, we compared the speech quality with the ECoG signal-based synthesized monosyllabic English words by Angrick et al. (2019). Then we used the MOS evaluation method and distributed questionnaires, inviting native Chinese speakers to score our synthesized speech from a subjective perspective.

We randomly selected six speech recordings from the training set for each word of each subject and set one of them as the reference speech, and the other five speech recordings were used to compare with the five synthesized speech. The speech quality of the synthesized speech is demonstrated by longitudinally comparing the recorded speech with the reference speech and the synthesized speech with the reference speech.

**FIGURE 9:**
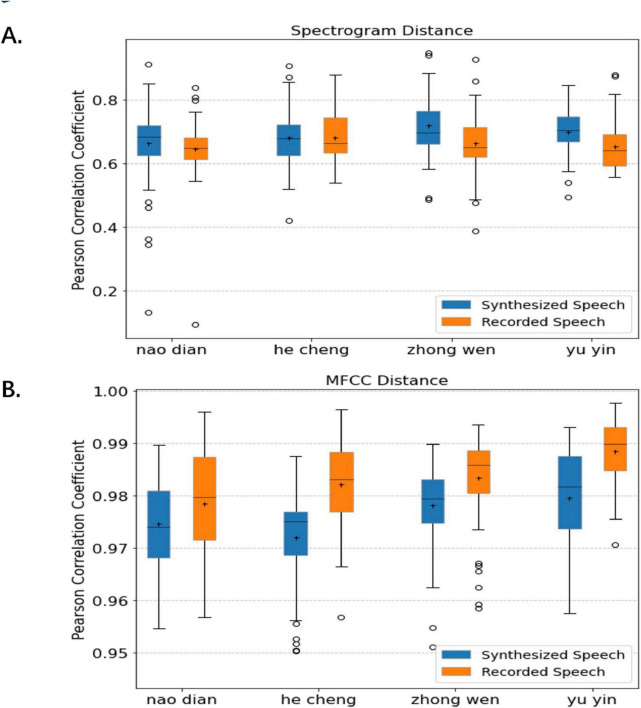
Comparison of the quality of synthesized speech in spectrogram **(A)** and MFCC **(B)** similarity. We plotted separately, for each word, the average similarity by all subjects of the imagined speech synthesized to the reference speech on the spectrogram and MFCC and the average similarity of the recorded speech to the reference speech on the spectrogram and MFCC.

As shown in Figure 9A, our synthesized speech is in significant agreement with the recorded speech in terms of the spectrogram distance to reference speech in different words, which reflects that we effectively maintain the Chinese pronunciation variability between words. Both have good performance in terms of the mean and extreme values of the intra-class similarity of the same word, which indicates that the accuracy gap between the synthesized speech and the reference speech is consistent with the level of the recorded speech, with credible accuracy. Additional notes, Since our reconstructed speech is corrected for frame order with the reference speech by the DTW algorithm, it performs slightly better than the recorded speech in terms of direct spectrogram distance. Table 2 gives the average spectral similarity of each subject across words. It can be seen that our synthesized speech is significantly more similar to the reference speech than the recorded speech (8/13).

**TABLE 2:** Spectrogram similarity results across subjects.

	Class	Nao dian	He cheng	Zhong wen	Yu yin	Average
Sub 1	Synthesized	0.59	0.69	0.71	0.62	**0.65**
	References	0.60	0.56	0.60	0.63	0.60
Sub 2	Synthesized	0.67	0.72	0.84	0.74	**0.74**
	References	0.63	0.71	0.70	0.65	0.67
Sub 3	Synthesized	0.69	0.64	0.67	0.69	**0.67**
	References	0.61	0.62	0.59	0.61	0.61
Sub 4	Synthesized	0.59	0.59	0.62	0.72	0.63
	References	0.66	0.67	0.64	0.66	**0.66**
Sub 5	Synthesized	0.61	0.65	0.74	0.69	**0.67**
	References	0.60	0.62	0.65	0.64	0.63
Sub 6	Synthesized	0.72	0.69	0.71	0.69	**0.70**
	References	0.67	0.70	0.70	0.63	0.68
Sub 7	Synthesized	0.63	0.57	0.68	0.71	0.64
	References	0.65	0.65	0.69	0.67	**0.66**
Sub 8	Synthesized	0.65	0.68	0.68	0.71	**0.68**
	References	0.53	0.59	0.54	0.62	0.57
Sub 9	Synthesized	0.68	0.67	0.73	0.71	**0.70**
	References	0.62	0.61	0.64	0.61	0.62
Sub 10	Synthesized	0.71	0.70	0.73	0.70	**0.71**
	References	0.65	0.66	0.67	0.65	0.66
Sub 11	Synthesized	0.57	0.69	0.77	0.68	0.68
	References	0.66	0.69	0.69	0.67	0.68
Sub 12	Synthesized	0.72	0.75	0.70	0.68	0.71
	References	0.73	0.72	0.72	0.75	**0.73**
Sub 13	Synthesized	0.74	0.76	0.71	0.7	0.73
	References	0.75	0.71	0.73	0.76	**0.74**

Bold text indicates which speech quality is higher for each subject.

**FIGURE 10:**
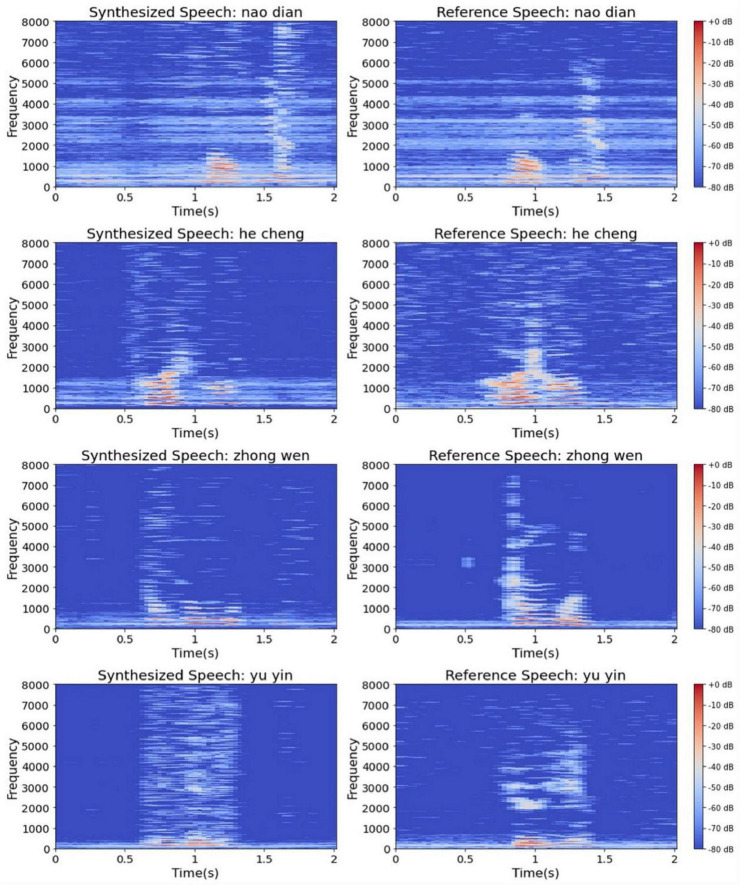
Spectrogram comparison of synthesized speech (left) and reference speech (right). The left column from top to bottom are the spectrograms of synthesized speech, the right column from top to bottom are the spectrograms of reference speech in that order.

The synthesized speech also has extremely high similarity to the reference speech in MFCC features, and the worst similarity is higher than 0.95 (see Figure 9B). The similarity distribution between the synthesized speech and the recorded speech is also similar, which indicates that the human ear audibility of our synthesized speech is high. It is worth stating that the monosyllabic audible speech synthesized by Angrick et al. based on ECoG has a similarity mean of 0.69 for the same calculation. This provides additional evidence that the quality of our synthesized speech from EEG is higher. Furthermore, to directly show the difference between our synthesized speech and the reference speech, we plotted the spectrograms of some synthesized speech and the corresponding reference speech, as shown in Figure 10. Table 3 gives the average MFCC similarity for each subject across words. It can be seen that the MFCC similarity between our synthesized speech and the reference speech is not as high as the recorded one, but the difference between the two is less than 0.2, and they are both higher than 0.97 overall.

**TABLE 3:** MFCC similarity results across subjects.

	Class	Nao dian	He cheng	Zhong wen	Yu yin	Average
Sub 1	Synthesized	0.98	0.97	0.98	0.99	0.98
	References	0.99	0.97	0.99	0.99	0.98
Sub 2	Synthesized	0.98	0.97	0.98	0.99	0.98
	References	0.99	0.98	0.99	0.99	**0.99**
Sub 3	Synthesized	0.97	0.98	0.99	0.99	0.98
	References	0.97	0.98	0.99	0.99	0.98
Sub 4	Synthesized	0.97	0.96	0.97	0.98	0.97
	References	0.98	0.99	0.98	0.98	**0.98**
Sub 5	Synthesized	0.98	0.98	0.98	0.99	0.98
	References	0.97	0.98	0.99	0.99	0.98
Sub 6	Synthesized	0.97	0.95	0.97	0.96	0.96
	References	0.98	0.98	0.96	0.98	**0.98**
Sub 7	Synthesized	0.97	0.97	0.97	0.98	0.97
	References	0.99	0.99	0.98	0.99	**0.99**
Sub 8	Synthesized	0.97	0.97	0.97	0.98	0.97
	References	0.98	0.99	0.99	0.99	**0.99**
Sub 9	Synthesized	0.98	0.97	0.98	0.98	0.98
	References	0.97	0.98	0.99	0.99	0.98
Sub 10	Synthesized	0.97	0.98	0.98	0.97	0.98
	References	0.96	0.97	0.980	0.99	0.98
Sub 11	Synthesized	0.97	0.98	0.98	0.97	0.98
	References	0.97	0.98	0.98	0.99	0.98
Sub 12	Synthesized	0.97	0.97	0.98	0.97	0.98
	References	0.99	0.98	0.98	0.99	**0.99**
Sub 13	Synthesized	0.97	0.97	0.98	0.99	0.98
	References	0.99	0.99	0.99	0.99	**0.99**

Bold text indicates which speech quality is higher for each subject.

**FIGURE 11:**
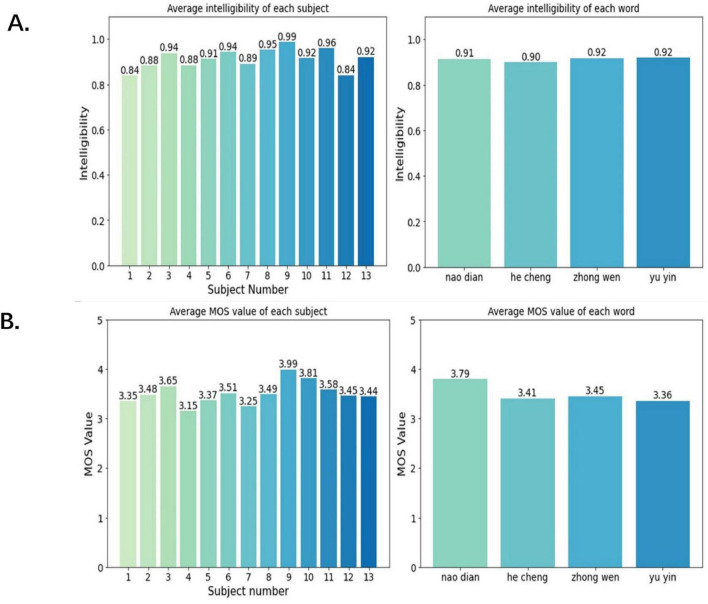
The results of synthesized speech in intelligibility **(A)** and MOS value **(B)**. The left graphs shows the average intelligibility and MOS value of the synthesized speech of each word. The right graph shows the average intelligibility and MOS value of the synthesized speech of each subject.

The subjective aspect was carried out in the form of a questionnaire by inviting 24 native Chinese speakers to select the words they heard from a list of four words after listening to the speech scoring the speech according to clarity and intelligibility. The scoring criteria for MOS value are shown in Table 4. All the synthesized speech data were presented to the respondents in a random order, a total of 2,715 pieces of valid evaluation data were collected and the results of the questionnaires are shown in Figure 11.

**TABLE 4:** MOS value scoring criteria.

Grade	Score	Listening experience
Excellent	5	Hear clearly, noiseless
Good	4	Hear clearly, a little noise
Fair	3	Can’t hear very well, understandable
Poor	2	Can’t hear very well, Need to repeat multiple times
Bad	1	Can’t understand

As shown in Figure 11A, the overall average intelligibility of each word exceeded 90%, and the highest intelligibility was 99% for subject 9, which indicates the high intelligibility of our Chinese speech synthesized by imagined speech EEG. The overall MOS value of each word exceeded the passing line (value three) of speech synthesis quality as seen in Figure 11B, and the MOS value of subject 9 even reached 3.99, which indicates that our synthesized speech has a good performance in clarity.

**FIGURE 12:**
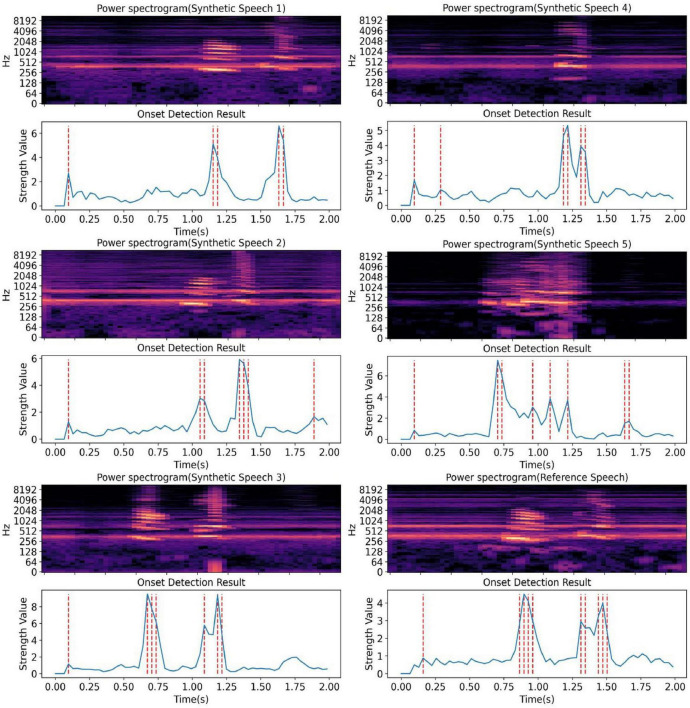
The onset detection results of synthesized speech (“nao dian”) from the same subject. We show the power spectrograms and audio rhythm graphs of five synthesized speech and one reference voice from the same subject on the same word. It can be seen that the rhythms of our synthesized speech are different from each other.

Since human pronunciation does not strictly follow grammatical structures, rhythm is a speaker-specific characteristic (Scarcella and Oxford, 1994). Rhythm has an important impact on the naturalness and intelligibility of speech synthesis. It divides the utterance into segments according to different tones by the natural breathing of humans, which enhances the rhythm and fluency of the utterance and also facilitates the elimination of some ambiguities. We plotted the power spectrogram and audio rhythm maps of the word “nao dian” synthesized from the same subject’s imagined speech EEG synthesis (locating note onset events by picking peaks in an onset strength envelope), as shown in Figure 12. The variability observed in Figure 12 reflects the subject-specific pronunciation rhythm, demonstrating that our end-to-end speech synthesis method effectively preserves the individual rhythm patterns of imagined speech. The differences are not due to model inconsistencies but rather an inherent characteristic of personalized speech synthesis. And considering the integrity of the syllables of our synthesized speech has been examined from both objective and subjective perspectives. Therefore, it can be said that our synthesized speech effectively preserve the rhythm of the subject’s imaginary pronunciation.

**FIGURE 13:**
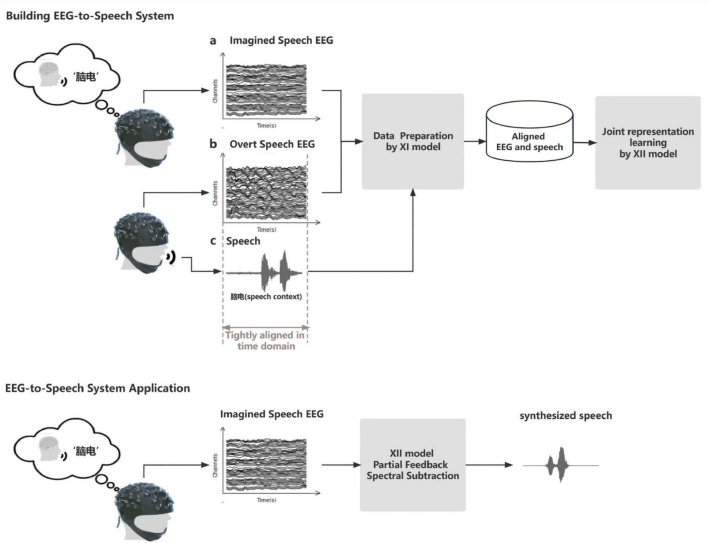
The framework of the EEG-to-Speech system. **(a)** EEG waveform of overt speech, **(b)** EEG waveform of imagined speech, and **(c)** speech waveform collected from the same trial. The system is trained in two steps. Step 1: The overt speech EEG is noise-reduced and aligned with the imagined speech EEG by the XI model. Based on the natural alignment of the overt speech EEG and speech, two time-matched data pairs are obtained: {imagined speech EEG, speech} and {denoised overt speech EEG, speech}. Step 2: The EEG and speech data pairs are fed into the end-to-end XII model to learn the mapping relationship between the two modalities. When the system is applied, the imagined articulatory EEG is sent to the XII model to obtain the synthesized speech.

In summary, it can be said that the speech decoded from the EEG based on imagined Chinese pronunciation evoked in this paper is objectively consistent with the recorded speech in terms of accuracy and intelligibility. And the whole framework of the EEG-to-Speech system is shown in Figure 13.

## 4 Conclusion

In this study, we accomplish the first time applying neural activity of human imaginary pronunciation to synthesize high-quality intelligible natural speech, which is a great breakthrough. The breakthrough is mainly reflected in (1) We constructed an end-to-end multimodal model that decodes audible high-quality imagined speech directly from neural activity. (2) In this paper, we used the non-invasive EEG as neural activity measurement which was polluted by lots of artifacts. But it has greater portability and a wider range of applications. Moreover, the frequency band range of the EEG signal is much smaller than the frequency band range of speech, and it is extremely difficult to synthesize speech signals directly from EEG across modalities. (3) We proposed a novel experimental paradigm and cross-modal time-domain information matching method to solve the problem of imagined speech neural activity that doesn’t have aligned natural speech.

We succeeded in synthesizing four Chinese words containing seven vowels and eight rhymes, covering all four tones, and constructing eight different phoneme combinations. Moreover, the application conditions of our ETS system do not restrict the language of the synthesis speech, which makes our method highly scalable. In addition, we introduced partial feedback and removed redundant information methods in the synthesis process. This strategy can be applied to multimodal learning to effectively improve prediction performance.

Our research may facilitate the development of regaining verbal communication ability in paralyzed patients. The primary aim of this study is to use solely imagined speech EEG signals for speech synthesis, catering to applications targeted at individuals with aphasia. In the synthesis process, any muscular electromyography (EMG) activity associated with vocalization is excluded, enhancing the robustness of the approach. This deliberate exclusion poses a greater challenge, reinforcing the study’s focus on decoding speech solely from brain activity.

However, it must be said that we still have great room for improvement. While this study is currently limited to a small vocabulary of four disyllabic Chinese words, the model’s frame-to-frame waveform-level conversion mechanism is inherently independent of vocabulary size or language type. This design supports scalability to continuous speech and different languages without modifying the core architecture. Expanding to natural, continuous speech may introduce challenges such as phonetic variability and coarticulation effects, which primarily affect training complexity rather than the model’s feasibility. Future work will explore sequence-level constraints and attention mechanisms to enhance performance under these conditions. The system’s generalization to unseen subjects remains a challenge due to EEG signal variability across individuals. Future studies will focus on adaptive learning techniques and larger participant groups to enhance robustness and inter-subject consistency.

## Data Availability

The original contributions presented in this study are included in this article/supplementary material, further inquiries can be directed to the corresponding author.
